# Perceived Impact, Needs, and Resources of Grandparents of Children and Adolescents on the Autism Spectrum: A Qualitative Study

**DOI:** 10.1007/s10803-024-06537-6

**Published:** 2024-09-06

**Authors:** Sofía Baena, Lucía Jiménez, Sonia Bejarano, Victoria Hidalgo

**Affiliations:** 1 Department of Psychology, Universidad Loyola Andalucía, Seville, Spain; 2 Department of Developmental and Educational Psychology, Universidad de Sevilla, Seville, Spain

**Keywords:** Grandparents, Autism, Impact, Needs, Resources, Qualitative

## Abstract

Grandparents play different roles in families of children and adolescents on the autism spectrum. They are frequently engaged in caregiving tasks with the person on the autism spectrum, providing emotional and instrumental support to the family. However, despite their frequent involvement and the importance of their role in the family, there are few studies that address the experiences of these grandparents, particularly in the Spanish and southern Europe context. This study explores the impact and needs of having a grandchild on the autism spectrum and the resources that grandparents have and use to face the difficulties that arise. A semi-structured interview was carried out with 17 grandparents of children and adolescents on the autism spectrum. We conducted a coding reliability thematic analysis of the impact and used a quantitative content analysis of grandparents’ needs and resources. Results indicated three main aspects related to the impact: personal growth, wanting to help and not being able to, and suffering at three levels: for themselves, their sons and daughters, and grandchildren. Grandparents perceived needs in four contexts: their own needs, the needs of the nuclear family, the needs of the person on the autism spectrum, and the needs of society. The most frequent needs were informational and management of behavioral difficulties. In the resources, the most frequently used strategies were religious beliefs and informal support seeking. It is essential to address the quality of parents-grandparents’ relationships, and include grandparents in intervention programmes, as a way of addressing grandparents’ needs.

Autism spectrum disorder (ASD), defined as difficulties in social communication and a restricted or repetitive pattern of behavior (APA, 2013), has an impact on the family. Studies indicate that parents of individuals on the autism spectrum present higher levels of stress and adjustment difficulties in comparison with parents of neurotypical children (Hayes & Watson, 2013; Ni’matuzahroh et al., 2022; Pastor-Cerezuela et al., 2021) or children with other disorders (Estes et al., 2009; Hayes & Watson, 2013; Pastor-Cerezuela et al., 2021). These studies have mainly focused on the nuclear family. However, members of the extended family, particularly grandparents, play an important role in the family and are also influenced by having a child or adolescent on the autism spectrum in the family. Particularly in the Spanish context studies have mainly focused on parents of individuals on the autism spectrum, particularly on the adaptation, stress, and positive perceptions of both mothers and fathers, using a quantitative approach (e.g., García-López et al., 2016; Pozo & Sarriá, 2015; Sarriá & Pozo, 2015). There are also some studies approaching the needs of the parents of individuals with ASD (e.g., Murillo & Belinchón, 2006) indicating particular needs of support for parents in respite care, continuity of support, and more professionalized support for their children. To our knowledge, there is only one study in Spain considering the general experience of grandparents with a grandchild with disabilities (Velasco et al., 2016). Using a qualitative approach, this study addresses the experience of grandparents who have a grandchild on the autism spectrum in the Spanish context.

The role of grandparents has become particularly relevant in the last decades, with an increase in the involvement of grandparents in the daily caregiving of children (Buchanan & Rotkirch, 2018; Osuna, 2006; Ponce et al., 2011). There are numerous ways of being a grandparent, with a wide range of factors influencing this experience (Buchanan & Rotkirch, 2018; Ramos, 2019). In general, studies indicate that involvement in grandchild caring is associated with higher levels of well-being and quality of life for all grandparents (Arpino et al., 2018; Leimer & van Ewijk, 2022), except when childcare is a daily occurrence (Leimer & van Ewijk, 2022). There is variability in how grandparents experience their grandparenting role, depending on how relevant this role is for them and their overall satisfaction with the role (Lozano-Sufrategui et al., 2022). Grandmothers are often more involved in grandchildren's caregiving practices than grandfathers (Ramos et al., 2021). Grandparents have a particularly relevant role in only-father families (Ramos et al., 2021), and in countries where familism is a central cultural value. Familistic values involve the extended family as one of the most important sources of social support (Badenes & López, 2011), and less involvement from formal sources of support for childrearing tasks (Calzada & Brooks, 2013; del Valle et al., 2013). Culture has an important role in shaping grandparent-grandchild relationships, and in how grandparenting is perceived and experienced (Hayslip et al., 2019). Thus, differences in cultural values could be expected to influence the grandparent-grandchild relationship (Hayslip et al., 2019). For example, in Spain, more than 80% of the population over 65 years old have daily contact with their sons, daughters, or grandchildren, and it is frequent for grandparents to live close to them (IMSERSO, 2018).

This support role is particularly relevant and complex in families of children and adolescents on the autism spectrum, as the support needs increase over time (DePape & Lindsay, 2015), and as such, their experience as grandparents may differ from other grandparents. Grandparents of individuals with disabilities have the potential to fulfill different roles within the family as a result of these family circumstances. Grandparents could have an important role in the detection of the first alarm signs related to difficulties in the development of children. Thus, frequent grandparent-grandchild interactions are related to a reduction in the delay of the diagnostic process due to their previous experience with the development of typically developing children as they have already been parents (Sicherman et al., 2018). They normally also play a role as a nexus between the family of the child on the autism spectrum and the rest of the extended family and could act as safeguards of the needs of typically developing siblings of people on the autism spectrum (Margetts et al., 2006; Milleret al., 2012; Prendeville & Kinsella, 2019). Lastly, among their most important and most studied roles, grandparents are frequently a source of emotional and instrumental support. Parents of children and adolescents on the autism spectrum consider grandparents as one of the most important sources of support, with an influence on the parents’ well-being (Crettenden et al., 2018; Divan et al., 2012; Fiske, 2017; Margetts et al., 2006; Trute, 2003; Trute et al., 2008; Velasco et al., 2016). Grandparents often provide economic support, take caregiving tasks when the parents are working and could act as a family respite option (D’Astous et al., 2013; Hillman, 2007; Hillman et al., 2016; Lee & Gardner, 2010; Novak-Pavlic et al., 2021; Prendeville & Kinsella, 2019; Trute, 2003). In addition, grandparents of children and adolescents on the autism spectrum are aware of the daily challenges mothers and fathers face (Sullivan et al., 2012; Yang et al., 2018) and often offer support through listening, helping with the search for possible solutions and the celebration of the small achievements of the child on the autism spectrum (Kaczmarek, 2021).

There is a heterogeneity in the grandparent-grandchild relationship of families with children on the autism spectrum, with different levels of involvement. Several studies have identified factors that can influence the involvement of grandparents in caring for children and adolescents on the autism spectrum. Firstly, there are gender differences in the level of implication, with grandmothers generally being more committed than grandfathers (D’Astous et al., 2013; Trute et al., 2008), and often a higher involvement from the maternal than the paternal side of the family (D’Astous et al., 2013; Hillman et al., 2016). Secondly, the physical closeness of grandparents from the family home is an important factor, with grandparents who live nearer being generally more committed and more frequently involved in daily caregiving tasks (D’Astous et al., 2013; Lee & Gardner, 2010). Another important factor is the quality of the grandparent-parent relationship, as parents act as gatekeepers for the children on the autism spectrum. Thus, low quality in this relationship is related to a lower probability of grandparental involvement (D’Astous et al., 2013; Lee & Gardner, 2010).

Few studies have focused on the impact of having a grandchild on the autism spectrum. Available evidence suggests that grandparents express feelings similar to parents, such as emotional ups and downs, increased levels of stress, and a high appreciation for the small achievements of the child on the autism spectrum (Fiske, 2017; Hillman, 2007; Hillman et al., 2016, 2017; Novak-Pavlic et al., 2021; Woodbridge et al., 2009). However, certain feelings and perceptions of the impact are idiosyncratic to grandparents. Some grandparents tend to prioritize the well-being of parents, and their supportive role, suppressing their own feelings after the diagnosis (Miller et al., 2012). These grandparents frequently perceive a double burden, understood as their involvement in caring for their grandchild and the parents, who are their own sons and daughters (Margetts et al., 2006), and often feel worried about the present and future of both the child and the nuclear family (D’Astous et al., 2013; Fiske, 2017; Hillman et al., 2016, 2017; Margetts et al., 2006). Also, they sometimes perceive their grandchild as an additional source of conflict between the family of the child on the autism spectrum and the extended family, particularly in terms of jealousy and anger about the unequal amount of attention to other grandchildren and the expectations from the family about how the interactions with the child with a disability should be (Miller et al., 2012).

At an individual level, grandparents sometimes perceive they have made sacrifices in their daily life and economically, having to reorganize their daily routines (Miller et al., 2012; Prendeville & Kinsella, 2019), and often contributing to the payment of therapies and treatments (Hillman et al., 2016). However, grandparents also perceive the relationship with their grandchild on the autism spectrum as an incentive to remain active, giving a sense of meaning to their lives (Hillman et al., 2017), and generally reporting good adjustment levels and resilience, despite the difficulties (Hillman et al., 2016).

The impact of having a grandchild on the autism spectrum involves specific needs in grandparents. This is an under-researched topic, although available evidence indicates that the most frequent needs perceived by grandparents are related to informational needs, learning how to communicate with professionals (Prendeville & Kinsella, 2019; Zakirova et al., 2020), and the management of disruptive behaviors (Zakirova et al., 2020), as not knowing how to face those situations could lead to anxiety and refusal to participate in the caring of the grandchild on the autism spectrum (D’Astous et al., 2013; Hillman et al., 2017). Furthermore, grandparents often express the need for recognition of their supportive role by professionals (Prendeville & Kinsella, 2019).

To support grandparents of children on the autism spectrum, it is important to delve not only into the impact and needs reported by grandparents but also into the resources that grandparents have to face these difficulties. From our knowledge, this topic has not been explored in recent literature, except the study of Hillman et al. (2016), who reported that grandparents from the USA, facing difficulties with their grandchild on the autism spectrum, search for support from different sources, both informal (e.g., their religious or support groups) and formal (e.g., psychologists or social workers).

To sum up, there has been an increase in the importance and involvement of grandparents of children and adolescents on the autism spectrum in caregiving tasks, particularly in countries with familistic values (Badenes & López, 2011). However, to our knowledge, few studies have approached the experiences, needs, and resources used by grandparents of children on the autism spectrum, with no studies being carried out in Spain or Southern Europe. The available studies highlight that there are shared elements of the experience between parents and grandparents, such as the emotional ups and downs and the celebration of small achievements (Fiske, 2017; Hillman, 2007). However, grandparents also have an idiosyncratic experience, such as the perception of a double burden (Margetts et al., 2006), which leads to specific support needs. The main needs are related to information and knowledge about the management of disruptive behaviors (Prendeville & Kinsella, 2019; Zakirova et al., 2020). Along with these needs, grandparents use resources when facing difficulties related to their grandchild on the autism spectrum, particularly in the form of support searching (Hillman et al., 2016). However, there are few studies about the resources and effective strategies that grandparents of children on the autism spectrum use, which are key elements in the design of useful and specific supports targeting this population.

The general aim of the present study is to explore the experiences of grandparents of children on the autism spectrum in the Spanish context. The research questions are (1) How does having a grandchild on the autism spectrum influence grandparents?; (2) what are the perceived needs of grandparents with a grandchild on the autism spectrum?; and (3) what are the self-identified resources grandparents use to face difficulties related to their grandchild on the autism spectrum?

## Method

### Participants

This study was carried out with a sample of 17 grandparents of children and adolescents on the autism spectrum. All participants had Spanish nationality, were retired or not working and lived in the same region as the child on the autism spectrum. We did not use the data saturation criteria to establish sample size, due to the use of an inductive method for coding, which made it hard to establish a sample size and use data saturation as a criterion (Braun & Clarke, 2021). Regarding their socio-demographic profile, the mean age of the participants was 69.67 years (*Me* = 71.50, *SD* = 7.66), and the age range was between 53 and 81 years. Most of the participants were maternal (76.47%) grandmothers (82.35%). Concerning their education level, most of them had completed secondary education (51.70%), followed by primary education (28.60%), whereas a small percentage had no studies (7.10%) or had completed higher education (7.10%).

Concerning the nuclear family of the child on the autism spectrum, most were biparental families, where both parents were living together (91.70%). In half of these families (54.54%) at least one of the parents was not working at the moment. The nuclear family had a mean number of children of 2.00 (*Me* = 2.00; *SD* = 0.74), with 25% of them having only one child. Parents of the children on the autism spectrum were a mean age of 43.17 (*Me* = 45.00, *SD* = 4.71), with an age range between 31 and 51.

The grandchildren on the autism spectrum were a mean age of 8.06 years old (*SD* = 3.07), with an age range between 2 and 15, more than half were male (64.71%), and half of them were the firstborn in their families (50.00%) Using as a reference the Childhood Autism Rating Scale (CARS; Schopler et al., 2002) filled by professionals working with the individual on the autism spectrum, 33.30% of individuals were rated as manifesting mild autistic characteristics, while 66.70% manifested moderate characteristics. To determine the severity, this scale measures the relationship with people, imitation, sensory responses, use of objects, adaptation to changes, verbal and non-verbal communication, activity level, and consistency of intellectual response. It does not take into account the presence of behavioral problems or other comorbidities.

### Instruments

#### Socio-demographic Profile Questionnaire

A questionnaire was developed ad hoc to gather information from the participants about their nationality, sex, age, professional situation, and participation in associations related to the scope of ASD. The administration of the questionnaire required approximately five minutes.

#### Semi-structured Interview with Grandparents of Individuals on the Autism Spectrum

An ad hoc semi-structured interview was conducted, organized around three content sections. The systemic perspective and the strengths approach were the theoretical basis of the interview; therefore, we focused on the relational dimensions, as well as on the resources and strategies grandparents found useful, besides the difficulties. The content sections were decided after a literature search about the experiences of grandparents of children on the autism spectrum, considering the research questions in the study; the general contents that were found were needs, coping strategies, relationship with the grandchild on the autism spectrum and impact on their life. The topic guide used in the study by Jensen et al. (2021), in which part of the research team participated, was used as a very initial guideline, and then included topics and contents based on the experience of the research team with interviewing families. One of the researchers made a proposal of multiple topics that could be included, and were later refined, transformed, and organized into specific questions with the rest of the research team.

The first section of the interview consisted of a set of introductory questions aimed at obtaining information about the relationship between grandparents and their grandchildren on the autism spectrum. Then, the participants were asked about the impact of having a grandchild on the autism spectrum on their lives. The third section of the questionnaire aimed to gather information about the support received and valued as useful..The total time required to carry out the interviews ranged between 13 and 57 min (*M* = 21.79, *SD* = 11.53). The complete interview protocol is available in Appendix I.

### Procedure

We conducted a cross-sectional study, using single-moment semi-structured interviews. We used a convenient and referral sampling strategy. Firstly, we contacted associations, schools, and intervention centers from five regions in the south of Spain, informed them of the study, and sent them an information document. Professionals from those centers would contact the families first and after they consent to participate, they would send the contact details to the research team. Once the family was included in the study, we interviewed the primary caregivers. After the interview with the primary caregivers, they referred the researchers to the grandparents. Inclusion criterion was that grandparents had to have a relevant and regular involvement in the life of the child on the autism spectrum, either face-to-face or by phone. The exclusion criteria were being physically or cognitively dependent and having any type of neurodegenerative illness such as dementia. All contacted grandparents agreed to participate in the study. We conducted the interviews at the time and location that the grandparents decided, according to their availability (home or intervention center). The interviewer was a psychologist trained in clinical psychology and with experience in interviews and focus groups. Afterward, we scheduled an appointment with the grandparents for the interview. Before starting the interview, grandparents were asked to sign a consent form and give verbal consent to record the interview. All grandparents agreed to audio record the interviews.

Ethical standards were followed according to the Helsinki Declaration, where the objectives and goals of the study were explained, along with the voluntary nature of the study, the anonymous use of the data, and their right to withdraw from the study and request exclusion of their data at any time without loss of benefits to which they were otherwise entitled. This study was approved by the regional ethics committee (1717-N17).

### Data Analysis

To respond to the different objectives of this study, we performed a thematic analysis, using a coding reliability approach, as defined by Byrne (2022), which allowed us to explore the impact perceived by grandparents. This approach to thematic analysis allows for capturing meaning via topic summarizing, and it emphasizes procedures for ensuring reliability and accuracy of coding (Braun & Clarke, 2023). In addition, we did a content analysis, which enabled the identification of the most frequent needs and coping resources, using the Nvivo software in both cases (v.12). Using a content analysis, we could identify the content or topics within the qualitative data and quantify the number of times they appear, along with interpreting the underlying context, as it is recommended when there is not much knowledge on the topic (Hsieh & Shannon, 2005). Means for establishing trustworthiness were discussed in each phase, following the examples reported by Nowell et al. (2017), who followed the criteria for trustworthiness from Lincoln and Guba (1985 as cited in Nowell et al., 2017).

The first step for each analysis was the familiarization with the data. For that, two of the members of the research team participated in the data collection, and the transcription of the interviews was done by a member of the research team and their subsequent verification by another member of the team. For the transcription process, we followed the recommendations by Braun and Clarke (2013). As a means to guarantee trustworthiness in this phase, we had a prolonged engagement with the data and stored raw data in well-organized archives (Nowell et al., 2017). All files with raw data and transcriptions were identified by a family identifier and relationship with the child on the autism spectrum and stored in organized folders. We also used an Excel sheet to detail the progress made by the team in the transcription and verification process. The second step was the creation of an initial code book containing the information suggested by Creswell and Poth (2018): a name for the code, a description of the code, and inclusion and exclusion criteria, and an example of the code. The inclusion and exclusion criteria of the codebook described in which circumstances the information would be coded under that specific code (e.g., we would use the code religious beliefs if grandparents specifically mention that their faith has either been shaken or reinforced as a consequence of having a grandchild on the autism spectrum) and if there were circumstances when we would not use that code or the alternative code that could be used instead (e.g., if they mention religious beliefs just as a belief we would not code it, and if they mention it is something helpful, we would code it under strategies).

Two interviews were randomly selected, and they were inductively (the codes were generated from the data) and jointly coded by two researchers, naming each code, and conducting a first organization approximation. Then, this initial codebook was refined and reorganized. Once the initial codebook was created, the rest of the interviews were coded, using a complete coding approach, executing the coding independently in this case, and using a flexible approximation, in which new codes were incorporated whenever it was required (Creswell & Poth, 2018). Following the recommendations of Campbell et al. (2013), different strategies were applied to facilitate the degree of agreement between the coders. First, we unified the units of meaning between the coders, which involved the more experienced coder deciding the parts of the transcript that were considered as fitting together in terms of meaning (e.g., sometimes they were three lines or one paragraph, depending on the meaning). Secondly, while coding, we incorporated definitions of the codes that were more specific, adjusting the inclusion and exclusion criteria to facilitate the inter-coder’s agreement. Lastly, the inter-coder’s agreement was computed, obtaining 62% agreement in the first interview and evolving until reaching 92% in the last interview. As a validation strategy, throughout the entire coding process, frequent meetings (4 in total) were held with another two members of the team for a peer review of the data and research process (Creswell & Poth, 2018). Concerning trustworthiness, and particularly credibility and dependability, we used researchers’ triangulation, a coding framework, peer debriefing, and an audit trail of code generation (Nowell et al., 2017). To sum up, as ways of carrying out researchers’ triangulation, two different researchers were coding independently initially and then came together to discuss a joint codebook, for this discussion a third researcher was incorporated to the discussion. Several rounds of coding independently the same transcripts (consensus coding) and coming together to check the coding helped with the reliability process. Along with researchers’ triangulation, we used peer debriefings to ensure reliability.

**Fig. 1:**
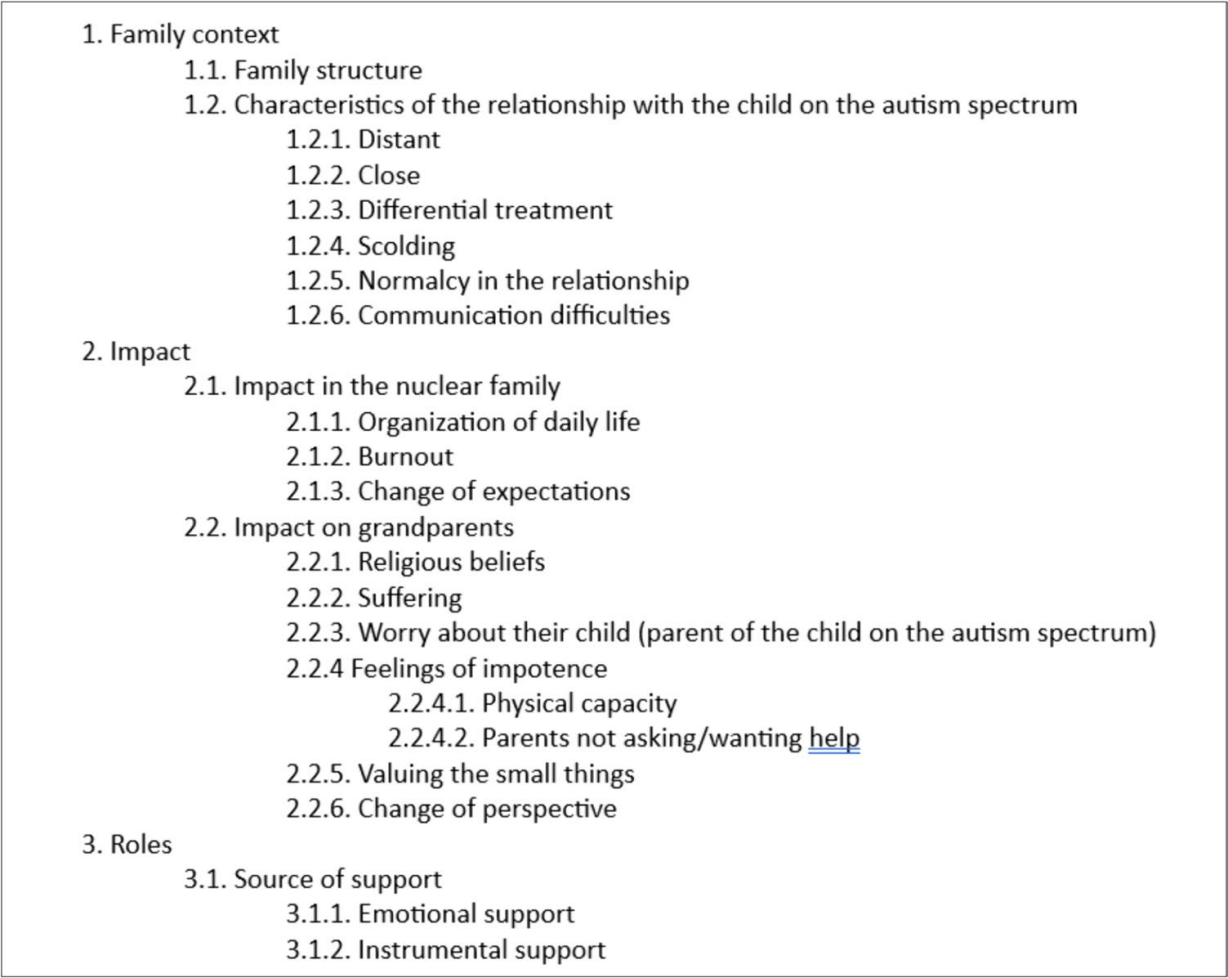
Fragment of final codebook

After executing these first steps, and to explore in more depth the impact of having a grandchild on the autism spectrum on the participating grandparents, we carried out a thematic analysis process. To this end, the different interviews were revised, making notes and relating the different codes, focusing on the relevant content and meanings, thus identifying the common themes and nuances related to the impact. These themes were derived from the final codebook, following the coding reliability approach to thematic analysis (Braun & Clarke, 2022). More specifically we used inter-coder agreement as a means to establish the reliability along with peer debriefings from other members of the research group. You can find a fragment of the codebook in Fig. 1. Four themes were initially generated, but after contrasting them against the data and a reflexive process, they were refined until obtaining the final three themes described in this study. Regarding credibility, the themes were triangulated by two different researchers and vetted by team members to reach a consensus. We have tried to describe in detail the process of coding and analysis, as to enable dependability (Nowell et al., 2017).

To study the needs and resources perceived by the participating grandparents of individuals on the autism spectrum, a quantitative approach to content analysis was performed, which allowed identifying the frequency of such needs and resources. Specifically, the code book was refined, grouping the codes into supraordinal categories that encompassed different contents. Then, we quantified the frequency with which the participants mentioned the different needs and resources, using the refined code book as a reference. This quantification was accompanied by a more precise explanation of each need and resource, as well as by an exemplary fragment of the participants’ testimonies. As the interviews were done in Spanish, we carried out the whole process in that same language. As a final step, for publishing purposes we translated the selected excerpts, the interview questions and the codebook to English. We had an English revision, and again a revision was made to ensure that the meaning and expressions of the excerpts was maintained.

## Results

### Impact of Having a Grandchild on the Autism Spectrum

The thematic analysis generated three relevant themes related to the impact of having a grandchild on the autism spectrum: *personal growth*, *wanting to help and not being able to,* and *suffering for others and oneself*.

#### Personal Growth

This first topic addresses the perceived positive consequences of having a grandchild on the autism spectrum. Participants expressed a positive perception toward having a grandchild on the autism spectrum, focusing on how sharing their daily routine with their grandchildren had helped them to better understand diversity and to be more patient and empathetic towards diversity. Moreover, some of the participants highlighted a special relationship with their grandchildren on the autism spectrum and the appreciation of small advances in developmental aspects that they did not consider to be relevant to their other grandchildren, such as saying a new word. Likewise, grandparents felt useful, and their daily routine gained meaning.

“I think it has been a positive impact, because... we have to make a greater effort to understand her (...) well, it has helped me to improve myself.” (Maternal grandmother of an 11-year-old granddaughter).

“Now he tells me ‘give me chocolate’, ‘give me round cheese’, ‘cheese’ (*they laugh*); he knows very well what he wants and I know too. So, I’m like ‘oh!’ We’re advancing a bit. Honestly, I get happy because of that. ‘Bye Grandma’ ‘Grandma’, that touches my heart.” (Paternal grandmother of a 9-year-old grandson)

Furthermore, it is relevant to highlight that grandparents perceived positive aspects in their relationship with their grandchildren on the autism spectrum and with the parents of the latter. The two main aspects highlighted by grandparents were the development of feelings of admiration toward their son or daughter in terms of the strengths and skills acquired, and their capacity to understand the person on the autism spectrum. Therefore, participants perceived personal growth in themselves and the parents of their grandchildren. Lastly, another positive aspect was the capacity to be strong when they thought they were overwhelmed, especially concerning the support provided to the parents of the person on the autism spectrum.

“So, I admire my daughter and my son-in-law, because I see they are much stronger than me.” (Maternal grandmother of a 6-year-old grandson)

#### Wanting to Help and Not Being Able To

The participants expressed feelings of impotence because, despite the desire to actively participate in the care of their grandchildren on the autism spectrum, in some cases, they could not provide such support. This feeling of impotence was frequently associated with the perception of greater needs for support and high levels of stress in the parents of children on the autism spectrum, since they perceived difficulties in their own daughter or son, and they could not help them overcome such difficulties. They identified two main barriers to supporting the parents of their grandchildren on the autism spectrum. Firstly, the parents behaved as gatekeepers; that is, despite their desire to help, parents did not ask for such help or did not want to leave their children on the autism spectrum under the care of their grandparents.

“I would love for him to be with me, to have him in my house at night so that his parents can go out and rest from their child, but…. Well, I speak with my daughter 20 times per day, over the phone or face to face, but alone with C, I only stay a little bit. She doesn’t even want me to stay…” (Maternal grandmother of a 6-year-old grandson)

The second barrier identified by the participants was related to their physical limitations since they stated that they could not take care of their grandchildren at a physical level, especially in those cases of individuals on the autism spectrum who presented disruptive behaviors. These situations generated feelings of impotence that involved, in many cases, suffering for not knowing how to act or help in those circumstances.

“(...) we didn’t know, I couldn’t do anything, so I told my R (mother of the adolescent on the autism spectrum) that, while the kid was like that, we couldn’t take care of him, which makes me sad... I got really depressed when I saw him hitting himself like that.” (Maternal grandmother of a 15-year-old grandson)

#### Suffering for Others and Oneself

The last theme referred to the suffering of the participants at three levels: for their son or daughter, for their grandchildren, and for themselves. Grandparents worried about themselves, their son or daughter and their families, and their grandchildren on the autism spectrum.

“Anyways, I think grandparents suffer twice as much for what happens to their grandchildren (...) Sure, I see the kid and I suffer, but I also see my daughter and I suffer too (...).” (Maternal grandmother of a 9-year-old grandson)

Part of the suffering of these grandparents was due to the misinformation and uncertainty they experienced about their grandchildren on the autism spectrum. In addition, they perceived that, in some cases, they could not have a similar relationship with them as they had with their other grandchildren. These difficulties in the relationship were sometimes caused by communication difficulties and the lack of understanding of the behaviors and needs of their grandchildren on the autism spectrum. As a response, they expressed feelings of confusion or irritation with God, believing that, as a family, they did not deserve the difficulties they were facing.

“Our relationship... well, he barely lets anybody kiss him; when we try to kiss him, he lowers his head... he doesn’t let us kiss him in the face or the head... he always keeps a distance…” (Maternal grandmother of a 7-year-old grandson)

“I got overwhelmed because I didn’t know, I didn’t understand that. There are no people with autism in my family. So, I said ‘Now what do we do? What school will we send him to? How are we going to treat him? And what *[worried tone]* … and what do we do with a little girl who doesn’t speak to us, and so it all came crashing down on me.” (Maternal grandmother of an 8-year-old granddaughter)

The main source of suffering reported by grandparents was related to the well-being of the parents of their grandchildren on the autism spectrum, since grandparents suffered from perceiving the burnout involved in the continuous care, not only at a psychological level, but also at a physical level, as well as the lack of time for leisure activities or to enjoy some free time, the economic impact, and the concern about the future.

“My daughter with moderate depression, both of us without a penny, the kid with those characteristics.” (Maternal grandmother of a 5-year-old grandson)

“Yes, that’s it, you have to understand both sides. As a grandmother, I love my granddaughter, but I also see my daughter suffering… so both things were mixed in my head.” (Maternal grandmother of a 9-year-old granddaughter)

Regarding the person on the autism spectrum, the main source of suffering was the observation of how the differences between the child on the autism spectrum and children the same age. This comparison caused them to face and adjust their expectations.

“I cried a lot every day. I thought about it all the time. Now, for instance, I would imagine him going out with girls at his age... feel very sad for him (...) his brother is flirting with girls, and I feel sad for him, cause he’s 15 years old and could be now... but no; oh, well.” (Maternal grandmother of a 15-year-old grandson)

Lastly, participants experienced suffering due to the concern about the future of the family, understanding that the person on the autism spectrum needed support and care that had been the sole responsibility of the parents up until this moment. The perception that their grandchildren on the autism spectrum would not achieve complete autonomy as adults, and the implications of this lack of future autonomy, both for the parents and the siblings, was a great source of concern for them. Regarding their future care, grandparents expressed their concern about the existence and adequacy of services targeting adults on the autism spectrum that could assume caregiving responsibilities when parents are no longer capable of performing them. Thus, participants manifested great concern about the lack of guarantees for the future care of their grandchildren on the autism spectrum.

“Bad, because I think of tomorrow. Honestly, I think of the future, because, if my daughter doesn’t have any more children, and my granddaughter has no siblings, well, I’m very worried about their future (…).” (Maternal grandmother of an 8-year-old granddaughter)

### Perceived Needs

Next, we present the results of the content analysis regarding the needs perceived by grandparents. A total of 12 categories were obtained, grouped into four supraordinal categories based on the scope in which the needs were perceived. Grandparents perceived their own needs (82.35%; *n* = 14), defined as needs related to their own role as a grandparent. They also widely identified needs related to the nuclear family (70.59%; *n* = 12), defined as the identification of needs related to the mothers and fathers of the person on the autism spectrum. Grandparents also identified needs related to the person on the autism spectrum (58.82%; *n* = 10) and needs related to society in general (35.29%; *n* = 6), defined as the changes that society must perform to guarantee adequate inclusion of individuals on the autism spectrum and their families. The needs perceived most frequently were their own needs and the needs related to the nuclear family of the person on the autism spectrum.

Next, for each context in which support needs were detected, we describe the specific needs identified, accompanied by the percentage and frequency of grandparents who detected such needs. Complementarily, we also present examples of their testimonies to illustrate each need.

#### Own Needs

Individual needs identified by grandparents are presented in Table 1, which included the need for support from professionals, at both the formative and psychological levels, as well as their need for greater involvement or participation in the lives of their grandchildren on the autism spectrum. Specifically, at the formative level, they expressed general training needs, related to obtaining information about ASD, and specific training needs in the management of disruptive behaviors focused on the learning of strategies of action in the face of rage or self-injury. In this sense, the most frequently perceived needs were those related to obtaining general training, focused on acquiring more information about ASD, followed by the need for specific training in the management of disruptive behaviors and the need to participate to a greater extent in the life of the person on the autism spectrum.

**Table 1:** Own needs perceived by grandparents of people on the autism spectrum

Needs	Description	% of grandparents who expressed the need (*n*)	Examples of testimonies
Formal training support:	Needs to receive greater training or information about ASD, to help them understand and take care of their grandchild on the autism spectrum	70.59 (12)	“Information (…) about the kid and… and how, how to treat him, uhm… some tools to be able to … Uhm, Uhm, interact with him”. (Maternal grandmother of a 5-year-old grandson) “And sometimes I don’t know… what to do, or say, or… anything, because (…) one day he started hitting himself… horrible (…) and we didn’t know, I couldn’t do anything.” (Maternal grandmother of a 15-year-old grandson)
General training		58.82 (10)	
Training in strategies for the management of disruptive behaviors		29.41 (5)	
Greater involvement or participation	Need to participate to a greater extent in the care of their grandchild on the autism spectrum, with a more active role in childcare support	29.41 (5)	“I would love him to be with me, to have him in my house in the night so that his parents can go out and rest from him.” (Maternal grandmother of a 6-year-old grandson)
Formal psychological support	Need to receive attention from psychologists, derived from the difficulties of having a grandchild on the autism spectrum	11.76 (2)	*“*Well, yes, some help would be great (…). Psychologically, yes, because it encourages us, which is what we need (…) because we want to help, but nobody helps us.” (Maternal grandmother of a 3-year-old granddaughter)

#### Needs of the Nuclear Family

Next, Table 2 presents the needs perceived by grandparents regarding the nuclear family of the person on the autism spectrum. Specifically, five main needs were detected: informal support, technical support, economic support, the need for leisure time, and the need for the parents to adjust their expectations. The most frequently identified needs were related to informal and technical support.

**Table 2:** Needs perceived by grandparents related to the nuclear family

Needs	Description	% of grandparents who expressed the need % (*n*)	Examples of testimonies
Informal support	Need of the parents to receive more support from the extended family	35.29 (6)	“He wishes his family paid more attention to him …” (Maternal grandmother of a 9-year-old grandson)
Technical support	Need of the parents to receive attention from psychologists, both at the emotional and formative level	35.29 (6)	“(…) My daughter needs to go to a psychologist.” (Maternal grandmother of an 8-year-old granddaughter)
Economic support	Need of the nuclear family to perceive some economic help to cover expenses derived from the needs of the personon the autism spectrum	17.65 (3)	“(…) because we don’t have that much money. And so, if the kid needs to go 1 day per week or 2 days per week, it could be 300, 350, 400 euros to have the kid there. Where do we get that money? (…).” (Maternal grandmother of a 2-year-old grandson)
Leisure time	Need of the parents to enjoy quality time as a couple and/or with their nuclear family	11.76 (2)	“(…) the kid can’t be taken to a place he doesn’t know. Obviously, that limits us very much, and if the parents don’t want to leave him with anybody, that’s it.” (Maternal grandmother of a 6-year-old grandson)
Adjustment of expectations	Need of the parents to start realizing that ASD is chronic and to accept reality regarding the person on the autism spectrum	5.88 (1)	“I think that his parents need to realize that the problem is real.” (Paternal grandfather of a 9-year-old grandson)

#### Needs of the Person on the Autism Spectrum

Regarding the needs of the children on the autism spectrum expressed by their grandparents, the only need detected was the lack of resources and, thus, the need of children on the autism spectrum to receive material and human resources that guarantee adequate care. Specifically, 58.82% of grandparents (*n* = 10) stated this need. Next, we show a fragment of a testimony that exemplifies this need.

“(...) a specialist who, well, does everything they can to make sure that the child develops, at least to help him learn to read. This would help me so much (...) some organization that at least makes us believe that there is a guarantee that he can live more or less happily and well, with his basic needs covered when his parents are gone (...)”. (Maternal grandmother of a 6-year-old grandson)

#### Needs Perceived in Society in General

Lastly, four grandparents (23.53%) referred to the need for raising awareness about ASD, thereby emphasizing the need for the general population to receive information and strategies to reduce prejudices and increase the understanding toward people on the autism spectrum and their families.

“Knowing this condition more; it should be shown more often on the TV, in the media, in the social networks, so that people become aware of what it is, of how these kids are.” (Maternal grandmother of a 7-year-old grandson)

With a lower frequency (17.65%; *n* = 3), the participants mentioned the need for professionals to be trained in this regard, for them to know and have strategies to work with children on the autism spectrum, thus improving the care and support they are provided.

“(...) teachers aren’t really prepared to attend a child with these characteristics, because they let them do whatever they want (...).” (Paternal grandmother of a 10-year-old grandson)

### Coping Resources Identified

Regarding the coping resources used by grandparents, 10 coping strategies and resources were identified and grouped into two supraordinal categories: cognitive-behavioral strategies and support-searching strategies. Grandparents used cognitive-behavioral strategies to a greater extent (94.12%; *n* = 16) than support searching strategies (58.82%; *n* = 10).

Next, for each type of resource identified, the specific strategies used by grandparents are described, accompanied by the percentage and frequency of grandparents who mentioned the use of such strategies. Complementarily, we present examples of their testimonies to illustrate each strategy.

#### Cognitive-Behavioral Strategies

The cognitive-behavioral strategies identified by grandparents are presented in Table 3. Religious faith was the main coping strategy, followed by positive thinking, acceptance, information searching, denial of the diagnosis, luck attribution, problem-centered strategies, and leisure activities.

**Table 3:** Cognitive-behavioral strategies used by grandparents

Strategies	Description	Grandparents who used the resource% (*n*)	Examples of testimonies
Religious belief	Understanding and acceptance of the situation based on religion	52.94 (9)	“(…) I truly believe in God, and if He sent him is because… that’s the way it had to be.” (Maternal grandmother of a 6-year-old grandson)
Positive thinking	Use of optimistic assertions focused on the strengths, that is, “seeing the glass half full”	35.29 (6)	“(…) like those who are born with a life-long malformation; however, in this case, the kid could get better (…).” (Maternal grandmother of a 7-year-old grandson)
Acceptance	Acceptance of their grandchild being on the autism spectrum	29.41 (5)	“(…) well, if this is what it is, it’s what it is… I don’t even think about it.” (Maternal grandmother of a 5-year-old grandson)
Information search	ASD-related information search	29.41 (5)	“I read a book (…) written by a mother who had a son with autism and… it helped me.” (Maternal grandmother of a 9-year-old grandson)
Denial of the diagnosis	Not accepting of their grandchild being on the autism spectrum right after the diagnosis	23.53 (4)	“(…) right now… uhm, I refuse to accept it… the diagnosis, you know? I don’t discard it, but… (…) But I don’t accept it either.” (Maternal grandmother of a 3-year-old granddaughter)
Luck attribution	Use of luck to explain why events occur	17.65 (3)	“I think we’ve been very unlucky. (…) obviously, there are other people with worse situations, but we have to deal with autism …” (Maternal grandmother of a 15-year-old grandson)
Problem-centred	Attention focused on solutions to solve the difficulties they encounter	17.65 (3)	“When she told me over the phone, she was crying (…) and I immediately said ‘look, we will find solutions. So, I called a couple of places…” (Maternal grandmother of a 6-year-old grandson)
Leisure activities	Performing activities that provide them satisfaction and relax	5.88 (1)	“(…) I often unwind from everything I have, by cross-stitching for instance. It helps me relax a lot.” (Paternal grandmother of a 10-year-old)

#### Support-Searching Strategies

Regarding support-searching strategies, grandparents identified mainly two sources of support, highlighting those of informal character. Thus, 58.82% (*n* = 10) of participants mentioned that they searched for support in their closer context, mainly resorting to the parents of the person on the autism spectrum as a source of information and support, rather than more professional or formal sources.

“(...) it’s difficult to make him understand better, although the kid has gone through many things and understands a lot, but I ask his mother anyways ‘What do we have to… [what do we have to do]What did they tell you? How do I get this?’ (...)” (Paternal grandmother of a 10-year-old grandson)

Only one grandmother (5.88%) mentioned searching for formal support, specifically from the professionals who work with her grandson on the autism spectrum. The type of support she had searched for in the formal scope was related to the acquisition of information and training.

“Well, thanks to the people in… in the CAIT [Early Intervention center] ... he is going once per week, (...) they are helping me a lot… to learn how certain things are done. (...) because they give you some guidelines, no matter how minimal they are.” (Maternal grandmother of a 5-year-old grandson)

## Discussion

This study aimed to explore the perceived impact of having a grandchild on the autism spectrum, as well as to identify the needs of grandparents and the resources they use to face the difficulties derived from the difficulties of their grandchildren in the Spanish context. Next, we discuss the obtained results based on these objectives.

### Impact on Grandparents of Children on the Autism Spectrum

Results of the present study have shown that having a grandchild on the autism spectrum has repercussions on the lives of their grandparents, with a diversity of experiences. In line with previous studies, this work reports that grandparents perceived positive aspects in the interaction with their grandchildren on the autism spectrum, such as greater appreciation of small achievements (e.g., the acquisition of different developmental milestones or verbal language; Fiske, 2017; Hillman & Anderson, 2019; Hillman et al., 2016, 2017). Another positive consequence was the perception of some grandparents of their grandchildren as significant individuals in their daily life, presenting their relationship with the person on the autism spectrum as a motivation to be active (Hillman & Anderson, 2019; Hillman et al., 2017). This process could be similar to grandparents of neurotypical children, as in general, grandparental involvement in childcare is associated with higher levels of well-being and quality of life (Arpino et al., 2018; Leimer & van Ewijk, 2022), and greater feelings of liveliness and purpose in life (Lozano-Sufrategui et al., 2022). A positive relationship with grandchildren has shown benefits that contribute to successful aging, such as the promotion of learning in grandparents in general (Ramos, 2019). We understand successful aging, according to the model of Rowe and Kahn (1987), as having a lower probability of developing diseases, enjoying good physical and mental functioning, and being actively committed to life. Thus, staying close to what provides a commitment to live is a key element in active aging (Paniagua, 2014). Having a grandchild on the autism spectrum, who requires more attention than a grandchild who is not on the autism spectrum, may lead grandparents to develop a more special relationship with their grandchildren and greater involvement in the family and other social relations. Thus, their grandchildren can become an important part of their daily life and possibly an element for their successful aging. Moreover, grandparents of children on the autism spectrum perceived feelings of overcoming. This could be explained by the resilience processes that have been identified in grandparents of children on the autism spectrum (Hillman et al., 2016, 2017), since the situation is valued as a challenge, as “something that God put in their way and which they can overcome”, instead of as a problem; therefore, by engaging in the care of their grandchildren on the autism spectrum, they experienced feelings of overcoming. This could be particularly relevant in the Spanish context, where religious beliefs are still prevalent among the elder population.

However, despite the perception of a positive impact, this study shows that having a grandchild on the autism spectrum also involves difficulties and concerns (Hillman et al., 2017). Specifically, grandparents felt sometimes impotent with the impossibility to help the child on the autism spectrum and particularly the parents and had negative feelings about their lack of involvement in the care of the grandchild on the autism spectrum. This is consistent with studies indicating that, in countries such as Spain, where grandchild caring support is expected from grandparents, not fulfilling this role is related to lower levels of satisfaction with well-being (Arpino et al., 2018), and possibly supports the idea that culture shapes how grandparenting is experienced (Hayslip et al., 2019). In the Spanish culture, where grandparents often live nearby and have daily contact with their sons, daughters, or grandchildren (IMSERSO, 2018), and have an important role in caretaking activities in normative circumstances, not being able to be involved in these tasks could be more negative than in other cultures.

Some of the reasons that grandparents identified as barriers to their involvement were their physical capacities. In some cases, these difficulties made it difficult or even prevented them from caring for their grandchildren on the autism spectrum, and as such prevented them from supporting the parents. This concern about physical deterioration or aging has also been reported in previous studies with grandparents of children on the autism spectrum (D’Astous et al., 2013; Prendeville & Kinsella, 2019) and in other studies with grandparents of children with disabilities (Miller et al., 2012). In this study, grandparents were worried about their physical capacities to a greater extent when their grandchildren presented disruptive behaviors, such as exhibiting aggression or tantrums, which could require physical strength to manage the situation. This result is consistent with their identified need for learning strategies for the management of these particularly challenging situations (D’Astous et al., 2013). Similarly, in this study, grandparents highlighted communication with the child on the autism spectrum as a possible barrier to a better relationship. When a person on the autism spectrum presents important difficulties in communication, the capacity to understand and attend to their needs might decrease, which could lead to stress in grandparents. Thus, the severity of ASD, particularly related to the presence of intellectual disability or communication disorders comorbidity could have more impact in the needs of grandparents. Finally, another barrier to the engagement of some grandparents was the reluctance of parents. A positive grandparent-parent relationship is related to more engagement in grandparents (D’Astous et al., 2013). Considering the importance of grandparents as sources of social support, identifying the motives and reasons behind parents’ reluctance, and promoting a positive grandparent-parent relationship could be important in these families (Hoang et al., 2022).

Results of this study indicate that grandparents could suffer due to their concern about the nuclear family of the person on the autism spectrum (Fiske, 2017; Hillman et al., 2016, 2017; Margetts et al., 2006), the well-being of their son or daughter (father or mother of the person on the autism spectrum) (D’Astous et al., 2013; Fiske, 2017; Hillman et al., 2016, 2017; Margetts et al., 2006) and the future of their grandchild when their parents would no longer be able to care for them (Miller et al., 2012; Prendeville & Kinsella, 2019). Likewise, some grandparents stated that they wished they had a more typical relationship with their grandchild, particularly referring to displays of affection and the need to spend more time together (Hillman et al., 2017). Frequent contact between grandparents and their sons, daughters, and grandchildren are common in the Spanish culture (IMSERSO, 2018), and as such, difficulties in the establishment of these relationships could have a bigger impact in this culture than in other countries.

### Perceived Needs

The results of this study have shown that grandparents perceived individual needs, as well as needs related to the family of the child on the autism spectrum and society in general.

The needs expressed by grandparents, such as the need for more formal and informal support, resources for the person on the autism spectrum, and the need to raise social awareness, are in line with the needs of parents of children on the autism spectrum reported in other studies (Derguy et al., 2015). This finding is not surprising, considering the studies that have highlighted grandparents as the main source of social support for parents of children on the autism spectrum (Findler, 2000; Fiske, 2017; Green, 2001; Heller et al., 2000; Margetts et al., 2006; Trute, 2003; Trute et al., 2008). Their valuable integration into family life could explain, in our opinion, the consistency with the needs of the parents found in other studies. This integration in family life, together with their capacity to detect alarm signals (Sicherman et al., 2018) and their role in perceiving and considering the needs of the siblings of children on the autism spectrum (Miller et al., 2012) shown in previous studies, pose valuable arguments to include them in the interventions designed for the family system.

The results of this study also indicate that, in our sample, grandparents did not perceive care as a burden, but as a need, and the fact that they could not be involved made them feel impotent, as they perceived the suffering and the needs of the parents and were not able or allowed to help. This result is in line with studies showing the positive relationship between involvement in the care of the person on the autism spectrum and greater psychological well-being in grandparents (Desiningrum, 2018). Specifically, grandparents pointed out the desire to be more involved in the lives of their grandchildren on the autism spectrum, thus helping the parents to enjoy some leisure time. The need to participate is in line with the findings of previous studies (Zakirova et al., 2020), however, it is important to highlight that most grandparents that participated in this study were maternal grandparents, who are generally more likely to be engaged in supporting the grandchild on the autism spectrum (D’Astous et al., 2013). Also, these results are consistent with the cultural context of the study. Spain is a country with familistic values, where involvement of grandparents is socially expected, and thus the lack of involvement could be related to lower levels of well-being in these grandparents (Arpino et al., 2018). Also, due to the frequent living proximity between grandchildren and grandparents, and the frequent contact between them (IMSERSO, 2018), their involvement could be easier than in other countries, and as such, the lack of involvement could be due to more relational reasons. Results also show the potential importance of addressing the possible barriers, difficulties, and fears in the interventions so that grandparents can be involved in the caregiving of their grandchildren on the autism spectrum. Among the barriers that were found in this study, the most important ones were physical difficulties, followed by difficulties in the management of disruptive behaviors, difficulties in communication, and the reluctance of the parents. These results are in line with their primarily perceived needs for general knowledge and training about ASD and disruptive behavioral management strategies, and consistent with other studies (e.g., Zakirova et al., 2020). Having a good understanding of ASD has a positive influence on the commitment of grandparents to the care of their grandchildren (D’Astous, 2013) and reduces their anxiety (Fortea et al., 2019). Thus, acquiring resources that help them to understand and manage difficult situations with their grandchildren could help them to maintain their involvement for a longer time, as well as learning alternative ways of communication with their grandchild on the autism spectrum. There are several parental training and education paradigms that have proven to be effective in reducing parenting stress, improving self-efficacy, teaching parents how to manage behavioral problems, and improving parent-to-child communication (Deb et al., 2020; Koegel et al., 2002; Vess & Campbell, 2022). This type of training could also be used with grandparents as caregivers.

In addition, incorporating information about what ASD is, general strategies, and pieces of advice on how to interact with a person on the autism spectrum and his/her parents are found among the contents of the interventions designed for grandparents (e.g., Fortea et al., 2019; Zakirova-Engstrand et al., 2021), and the specific guides developed by different entities (e.g., Autism Ponce & Vega, 2007; Speaks, 2013). Moreover, considering that another need perceived by grandparents was the need for greater involvement, training can be key to the promotion of such participation.

In contrast with the results obtained in this work, previous studies have reported that grandparents demand recognition for their contribution to the care of their grandchildren (D’Astous et al., 2013; Miller et al., 2012; Prendeville & Kinsella, 2019). However, this need was not found in the present study, since grandparents stated that they did the same with the rest of their grandchildren: taking care of them whenever they needed. This discrepancy could be explained due to cultural differences since the studies that highlight this need for recognition were conducted in the USA and Ireland. In Spain, a country characterized by familism as a cultural value, there is a high family commitment, with most adults over 60 years old considering the care of grandchildren as part of their daily grandparental role (Ayuso, 2012). Also, in Spain, due to grandparents living nearby (IMSERSO, 2018), taking care of grandchildren in general is an extended practice, and as such, it is not considered as something worthy of special recognition, but rather is socially expected.

### Resources Used

There is little research focused on studying the coping strategies used by older people (Rubio et al., 2019), and even scarcer evidence on the coping strategies used by grandparents with grandchildren on the autism spectrum. Therefore, this study poses a contribution to the resources and strategies implemented by grandparents of children and adolescents on the autism spectrum.

Results of this study indicate that most grandparents of children and adolescents on the autism spectrum used a wide variety of coping resources. While most grandparents used at some point avoidant strategies, (diagnosis denial and luck attribution), the most widely used coping strategies were active and positive, both emotion and problem-focused (religious belief, search for informal support, positive thinking, acceptance of the disorder, information search and search for formal support), as well as some avoidant resources to cope with this kind of situations. These results are consistent with those found in parents of persons on the autism spectrum (Lai & Oei, 2014). Although no studies have reported the efficacy of specific strategies in grandparents of children on the autism spectrum, previous studies with parents of individuals on the autism spectrum show that these types of active strategies are related to lower stress and anxiety levels, being thereby associated with better adjustment and thus more effective (e.g., Ekas et al., 2015; Ni’ matuzahroh et al., 2022).

One of the most important strategies used by grandparents in this study was religious belief. The study of Hillman et al. (2016), in agreement with our results, shows that relying on religious beliefs is a valuable resource for grandparents and the use of religious beliefs has been associated with higher levels of quality of life in the family life of parents of children on the autism spectrum (Boehm & Carter, 2019; Boehm et al., 2015; Brown et al., 2006). Results from our study indicate the importance of religious beliefs as coping strategies for grandparents in the Spanish context, in contrast with studies from other countries where grandparents did not perceive the need for support from religion or clergy (Hillman et al., 2016; Zakirova-Engstrand et al., 2020)This may be due to cultural issues, such as the still high relevance of Catholic religion for grandparents of children on the autism spectrum in Spain, similar to studies from several years ago in other countries, such as the USA (e.g., Vadasy et al., 1986), demonstrating that, apart from generational aspects, it is important to consider the cultural characteristics of each context.

Moreover, this study highlights the importance of informal social support for grandparents of children and adolescents on the autism spectrum. The search for informal support, particularly in the family context, emerged in this study as the most widely used resource by grandparents along with religious beliefs. Generally, grandparents are considered an important source of instrumental and emotional support for the families of children on the autism spectrum (D’Astous et al., 2013; Hillman et al., 2016; Prendeville & Kinsella, 2019; Trute, 2003). However, this study highlights that they are also recipients of support. Other studies have reinforced this understanding of grandparents as receivers of social support, indicating that older people use social support to face stressful situations, such as a partner’s death (Van Baarsen, 2002). The fact that the sample of this study was mostly constituted by women should be also considered, as other studies have shown that the search for informal social support is a strategy used preferentially by women (Navarro & Bueno, 2005).

It is also interesting to highlight that searching for formal support, which would include psychological support, was only used by one participant in this study. On the one hand, this is in contrast with the findings of previous studies (Hillman et al., 2016; Zakirova et al., 2020), in which the use of the resources of formal support such as psychological help was among the main resources used and needs detected by grandparents. This contrast may be due to methodological differences since not all studies have asked the participants about the same resources used; likewise, the use of qualitative methodologies hinders the comparison of studies in terms of higher or lower frequency of use. This discrepancy could also be due to cultural differences, in line with what was discussed in previous paragraphs concerning familism, where the bonds among the family members of different generations are very strong due to the cultural value of familism (Badenes & López, 2011; Calzada & Brooks, 2013; del Valle et al., 2013), and the structure of childcare service provision has a strong reliance on family childcare.

## Conclusions and Practical Implications

The results presented in this study shed light on the experience of a poorly studied population, particularly in the South European context: grandparents of children and adolescents on the autism spectrum. The methodological approach allowed us to analyze the experience of these grandparents in detail, thus enabling us to understand the complexity of such experiences and also detect perceived needs and strategies used by this population.

This study shows that having a grandchild on the autism spectrum has in general an impact on the lives of grandparents, with diversity in the experiences. Most grandparents identify positive aspects of having a grandchild on the autism spectrum alongside some difficulties or suffering, particularly experiencing a concern for the parents and their grandchildren. Moreover, this study indicates that grandparents potentially have a wide range of strategies and resources to cope with stressful situations derived from having a grandchild on the autism spectrum, highlighting religious beliefs and informal support as their main effective strategies, which could help them in turn to be a strong source of emotional and instrumental support for the nuclear family of the person on the autism spectrum.

This study is not without limitations. The sample of participants is limited to Southern Spain and with a wide age range of children on the autism spectrum and grandparents, thus these results must be generalized with caution. In addition, the diversity of the sample in terms of the phenotypical characteristics of the children on the autism spectrum was not considered. Future research could incorporate other measures of autistic phenotype, such as sensory difficulties as well as the presence of comorbidities. Moreover, there is a certain bias regarding the motivation of the participants, since the interviewed grandparents were involved in the care of their grandchildren on the autism spectrum, future studies should explore in a more specific manner the heterogeneity in the grandparent-grandchild relationship, including more information about proximity, frequency of care or type of care, for example. Moreover, grandparents were asked to participate by the parents of individuals on the autism spectrum, indicating a probable good relationship between them; they also participated voluntarily in this study. In addition, the sample was constituted of more grandmothers than grandfathers, and most of them were maternal grandparents. Also, most families were biparental and half of them had one parent staying at home. Also, the impact of the socioeconomic status and more in depth analysis of the influence of ASD severity could be relevant to include in future research. It could be interesting to see if these results could be generalized to other family structures and circumstances. As such, further quantitative studies should be carried out as to increase representativeness and make possible further comparisons with other countries. Finally, although some means for ensuring trustworthiness were used, more audit trail of the process, the use of reflective diaries, and a further description of the context could be helpful to improve trustworthiness, particularly transferability.

Despite of limitations, some implications of interest may be deduced. At the practical level, it would be important to address the parents-grandparents relationship, promoting effective communication strategies and delving into the barriers and fears related to grandparents as caregivers. With this improvement in communication between parents and grandparents, the aim would be for parents to further involve grandparents in the life of the minors on the autism spectrum, as well as to strengthen the reciprocal support between parents and grandparents, benefitting the whole family system (Hoang et al., 2022). One way of addressing these needs is including grandparents in the therapy sessions with the parents, or incorporating specific sessions where the parent-grandparent relationship is targeted. An example is a psychoeducational program for parents of children on the autism spectrum, which also includes a session where extended family members are invited. In this session, they focus on communication and sharing experiences from different points of view (García, 2020). Taking into account that parents act as gatekeepers of the child on the autism spectrum, the sharing of experiences between parents and grandparents, as well as generating exchange spaces between both, is important to break that involvement barrier. Similarly, this study shows the importance of developing intervention programmes in which grandparents are direct beneficiaries, or including them in adapted parent training programmes, as they have similar emotional reactions as parents, and have needs that could be addressed through these programmes. There are some recommendations (e.g., Ponce & Vega, 2007), experiences of workshops carried out by entities (e.g., Tea Cast, 2021), and intervention programmes aimed at grandparents that include the promotion of strategies for grandparents to learn how to support their grandchildren when they face different difficulties (Zakirova-Engstrand et al., 2021). From the results presented in this study, these programmes could incorporate not only information about ASD, but also provide grandparents with strategies such as disruptive behavior management tools and reinforce the resources they already have and use. Likewise, these programmes could potentially address emotional aspects, promoting the perception of efficacy of grandparents and tackling their concerns, providing them with strategies to help them. Specific policies to promote these approaches in programmatic interventions as well as for developing awareness campaigns on the role of grandparents for people on the autism spectrum could be enhanced. Finally, at a research level, this study reinforces the relevance of carrying out culturally contextualized research, as well as the importance of understanding the relationships as bidirectional and transactional. To keep progressing on this topic, future research should incorporate intra-family perspectives in the study of grandparents as to analyze in more depth the bidirectional relationships within the family. In addition, specific ways of promoting grandparental involvement and improving communication with the child on the autism spectrum could be examined.
